# The α–β phase transition in volcanic cristobalite

**DOI:** 10.1107/S160057671401070X

**Published:** 2014-06-14

**Authors:** David E. Damby, Edward W. Llewellin, Claire J. Horwell, Ben J. Williamson, Jens Najorka, Gordon Cressey, Michael Carpenter

**Affiliations:** a Department of Earth Sciences, Durham University, South Road, Durham DH1 3LE, UK; b Institute of Hazard, Risk and Resilience, Durham University, South Road, Durham DH1 3LE, UK; c Department of Earth and Environmental Sciences, Ludwig-Maximilians-Universität München, Munich 80333, Germany; d Camborne School of Mines, College of Engineering, Mathematics and Physical Sciences, University of Exeter, Penryn TR10 9EZ, UK; e Department of Mineralogy, Natural History Museum, Cromwell Road, London SW7 5BD, UK; f Department of Earth Sciences, University of Cambridge, Downing Street, Cambridge CB2 3EQ, UK

**Keywords:** cristobalite, phase transitions

## Abstract

Cristobalite is a common mineral in volcanic ash produced from dome-forming eruptions. Assessment of the respiratory hazard posed by volcanic ash requires understanding the nature of the cristobalite it contains. Volcanic cristobalite contains coupled substitutions of Al^3+^ and Na^+^ for Si^4+^; similar co-substitutions in synthetic cristobalite are known to modify the crystal structure, affecting the stability of the α and β forms and the observed transition between them. Here, for the first time, the dynamics and energy changes associated with the α–β phase transition in volcanic cristobalite are investigated using X-ray powder diffraction with simultaneous *in situ* heating and differential scanning calorimetry. At ambient temperature, volcanic cristobalite exists in the α form and has a larger cell volume than synthetic α-cristobalite; as a result, its diffraction pattern sits between ICDD α- and β-cristobalite library patterns, which could cause ambiguity in phase identification. On heating from ambient temperature, volcanic cristobalite exhibits a lower degree of thermal expansion than synthetic cristobalite, and it also has a lower α–β transition temperature (∼473 K) compared with synthetic cristobalite (upwards of 543 K); these observations are discussed in relation to the presence of Al^3+^ and Na^+^ defects. The transition shows a stable and reproducible hysteresis loop with α and β phases coexisting through the transition, suggesting that discrete crystals in the sample have different transition temperatures.

## Abstract 2

Volcanic cristobalite commonly contains structural substitutions of Al^3+^ and Na^+^ for Si^4+^. Quantifying the effect of these substitutions on the crystal structure may provide insight into volcanic processes and the variable toxicity of crystalline silica.

## 1. Introduction   

### 1.1. Background   

Cristobalite, a crystalline silica polymorph, forms in volcanic lava domes through vapour-phase mineralization and devitrification of groundmass (Baxter *et al.*, 1999 ▶; Horwell *et al.*, 2013 ▶). Volcanic domes are mounds of highly viscous lava that pile up over the vent rather than moving away as a lava flow. In these domes, metastable cristobalite is found in preference to quartz as its formation is kinetically favoured (Brodie & Rutter, 2000 ▶), and because of the large activation energy required for the reconstructive phase transition from cristobalite to quartz. Cristobalite is stable at high temperatures and low pressures (1743–2001 K at 1 atm; 1 atm = 101.325 kPa) in its cubic β form, and undergoes a spontaneous displacive phase transition to tetragonal α-cristobalite upon cooling. The β to α transition is accompanied by a ∼5% reduc­tion in volume, which causes the crystals to crack on cooling (Carpenter *et al.*, 1998 ▶); the resulting ‘fish-scale’ texture is indicative of cristobalite that has undergone the displacive transition.

As a result of the β to α transition, β-cristobalite is not expected in nature under ambient conditions. However, several X-ray diffraction (XRD) studies of cristobalite in volcanic rocks have identified both the α and β forms. For example, Keith & Muffler (1978 ▶) analysed cristobalite in tuff deposits in the Yellowstone caldera, USA, and found both α- and β-cristobalite. Keith *et al.* (1978 ▶) further identified β-cristobalite in hydrothermally altered rhyolite flow deposits at Yellowstone. More recently, Reich *et al.* (2009 ▶) observed polycrystalline aggregates of cristobalite nanofibres in ash erupted from Chaitén volcano, Chile. These were identified as β-cristobalite by diffraction pattern indexing of cubic lattice parameters *a* = *b* = *c* = 7.17 Å, characterized by the 111, 220, 331 and 422 reflections.

Volcanic domes are inherently unstable and prone to collapse. On collapse, the cristobalite-bearing lava fragments to produce cristobalite-rich ash, prompting concern for exposed populations (Horwell & Baxter, 2006 ▶). Crystalline silica is a known human carcinogen and long-term exposure may lead to silicosis, a chronic fibrotic lung disease (Beckett, 2000 ▶; IARC, 1997 ▶); consequently, much work has been carried out in recent years to establish whether volcanic cristobalite can cause disease (*e.g.* Jones & BéruBé, 2011 ▶; Wilson *et al.*, 2000 ▶; Horwell, Fenoglio *et al.*, 2003 ▶; Forbes *et al.*, 2003 ▶). Toxicological studies report low toxicity for cristobalite-bearing ash compared with α-cristobalite controls (*e.g.* Cullen *et al.*, 2002 ▶) in short-duration experiments; however, the toxicity of β-cristobalite is entirely unknown.

The biological reactivity of inhaled crystalline silica is governed by the origin and state (including whether freshly ground or aged) of the sample and its presence in a complex constitution, such as volcanic ash (Clouter *et al.*, 2001 ▶; Fubini *et al.*, 1995 ▶; Mossman & Glenn, 2013 ▶). As such, its pathogenic potential has been deemed a ‘variable entity’, whereby chemical and structural variability can modulate its toxicity (Donaldson & Borm, 1998 ▶; Napierska *et al.*, 2010 ▶). The role of the crystallization environment and mineralogy on the toxicity of volcanic cristobalite has only recently been considered: Horwell *et al.* (2012 ▶) reported the substitution of up to 2.4 mol.% Al_2_O_3_ for SiO_2_ in cristobalite from Soufrière Hills volcano, Montserrat, and suggested that this could potentially reduce its toxicity since Al has been shown to quell crystalline silica toxicity (Duffin *et al.*, 2001 ▶; Stone *et al.*, 2004 ▶). In cristobalite, Al^3+^ frequently occupies Si tetrahedral sites and Na^+^ occupies the interstitial nonframework sites for charge balance (Perrotta *et al.*, 1989 ▶; Thomas *et al.*, 1994 ▶). Nonspecific structural modifications have also been implicated in the bioreactivity of crystalline silica (Guthrie, 1997 ▶), although never considered for volcanic cristobalite.

In this work, we exploit the α–β phase transition to determine the effect of the substitutions described by Horwell *et al.* (2012 ▶) on the crystal structure, and compare the behaviour of volcanic cristobalite with the known structural and thermodynamic character of synthetic cristobalite. This is a necessary step towards understanding the formation and preservation of metastable cristobalite in volcanic systems. Additionally, since the physicochemical properties that govern the biological response to crystalline silica are directly related to the origin of the sample (Fenoglio *et al.*, 2000 ▶), this investigation furthers our understanding of the relationship between intrinsic mineralogy and the surprisingly limited *in vitro* toxicity of cristobalite-bearing volcanic ash.

### 1.2. The α–β phase transition in synthetic cristobalite   

The transition between the α and β forms of cristobalite is first order; hence, it is expected to be instantaneous and to correspond to a discontinuous jump in entropy (Schmahl *et al.*, 1992 ▶). However, many studies of synthetic cristobalite samples have shown that the transition occurs over a temperature interval of ∼20–40 K (Leadbetter & Wright, 1976 ▶; Mosesman & Pitzer, 1941 ▶; Sosman, 1965 ▶). This transition range exists because individual crystallites within a cristobalite-bearing sample may have considerably different transition temperatures depending on their size, structure and composition. Furthermore, a proportion of grains may persist metastably after they have passed their transition temperature. Consequently, at a given temperature within the transitional range, α and β forms coexist both stably and metastably (Leadbetter & Wright, 1976 ▶; Swainson *et al.*, 2003 ▶; Schmahl *et al.*, 1992 ▶; Spearing *et al.*, 1992 ▶). The α–β transition has been shown to depend on temperature, but not on time, demonstrating that the finite width of the transition range is not simply a kinetic effect (Schmahl, 1993 ▶; Leadbetter & Wright, 1976 ▶). A marked hysteresis is also consistent with the first-order character.

The α–β transition temperature in synthetic cristobalite is highly variable, ranging from 443 to 543 K (Frondel, 1962 ▶). This variability predominantly results from structural substitutions, along with the associated defects, vacancies and strains. Within this range, lower α–β inversion temperatures are associated with structural imperfections; for example, when a mineralizer [*e.g.* Al_2_(SO_4_)_3_ and Na_2_CO_3_] is present during crystallization (Chao & Lu, 2002*b* ▶; Stevens *et al.*, 1997 ▶). It is known that cation substitutions in cristobalite cause a change in the lattice dimensions, which results in a shift in the prominent 101 α-cristobalite peak position observed by XRD. Chao & Lu (2002*a* ▶) have shown that this displacement is proportional to the level of (Al_2_O_3_ + Na_2_O) co-doping during cristobalite synthesis; they find that *d*_101_ increases from 4.040 Å at 2.91 mol.% Al_2_O_3_–Na_2_O to 4.058 Å at 4.65 mol.%. These levels of doping are of the order of those seen for volcanic cristobalite (up to 2.4 mol.% Al_2_O_3_). The α–β transition may be suppressed entirely when substitution is extensive (*e.g.* Chao & Lu, 2002*b* ▶; Perrotta *et al.*, 1989 ▶). However, in the SiO_2_–Na_2_O–Al_2_O_3_ system, stabilization of β-cristobalite at room temperature was not observed below 6.29 mol.% Al_2_O_3_ in synthetic samples (Chao & Lu, 2002*a* ▶). The β to α transition in the closely analogous quartz system can also be suppressed by incorporation of other elements into the SiO_2_ structure: Xu *et al.* (2001 ▶) found that the high-temperature β form of quartz can be preserved down to room temperature through the substitution of Al^3+^ and Li^+^ for Si^4+^. In this case, suppression is due to a reduction in the transition temperature to below ambient temperature. The presence of foreign ions in the interstices and altered bond angles from Al coordination presumably inhibit the contraction of the structure.

The α–β transition temperature and transition interval may also depend on the crystallization and annealing history of the sample, since these influence crystallite properties. Lower transition temperatures are associated with lower crystallization temperatures; for example, Alcalá *et al.* (1996 ▶) observe a transition temperature of 523 K for SiO_2_ annealed at 1673 K *versus* 503 K when annealed at 1373 K. By carefully controlling the conditions of synthesis, it is possible to create cristobalite samples in which the α–β transition interval is very narrow. Thompson & Wennemer (1979 ▶) report a sharp peak in heat capacity at 535 K for a highly crystalline synthetic cristobalite (prepared at 1773 K for 20 h), indicating that a narrow transition range may reflect a homogeneous crystal population. Conversely, multiple phases of disordered growth and incorporation of impurity ions could increase the breadth of the transition interval (Stevens *et al.*, 1997 ▶).

The α–β transition temperature can also be a function of crystal grain size, which is dependent on duration of formation. In synthetic samples, increased annealing times lead to larger average grain sizes, although grain growth occurs heterogeneously (Lee & Lee, 2000 ▶). Analogously, in the quartz system, the thermodynamics of the α–β transition differ depending on the origin and size fraction of the sample; changes in the primary 101 quartz peak intensity that often accompany the α–β quartz inversion are a function of particle size (Wahl *et al.*, 1961 ▶). In general, large particles favour the spontaneous formation of low-temperature (α) phases, whereas small particles stabilize the high-temperature (β) phase (Lee & Lee, 2000 ▶), and phase transitions can become sensitive to grain size in nanoscale crystals owing to the relationship between atomic positions and macroscopic strain (Pertsev & Salje, 2000 ▶). Rios *et al.* (2001 ▶) show this for the transition from β to α quartz, but the change from normal properties to ‘nano’ properties, when the bulk properties become dominated by surface effects, occurs below ∼50 nm (McKnight *et al.*, 2008 ▶).

## 2. Materials and methods   

### 2.1. Volcanic ash and cristobalite reference samples   

Bulk ash sample MBA5/6/99 from Soufrière Hills volcano, Montserrat, West Indies, was analysed (Montserrat Volcano Observatory reference number MVO1163). It was collected during a dome-collapse event on 5 June 1999 and has been used extensively in ash characterization studies (*e.g.* Horwell, Fenoglio *et al.*, 2003 ▶; Horwell, Sparks *et al.*, 2003 ▶). The sample contains ∼11 wt% cristobalite (Horwell *et al.*, 2014 ▶).

Two cristobalite standards were analysed as controls: a pure-phase synthetic sample (denoted DKSmith) and an impure synthetic sample (denoted Hemenway). The samples were supplied by Deane K. Smith (Pennsylvania State University) and David Hemenway (University of Vermont).

### 2.2. Concentration of crystalline silica component   

The sub-4 µm ‘respirable’ fraction of MBA5/6/99 (denoted MRA5/6/99) was also analysed. It was separated from the bulk sample using a British Rema Minisplit Aerosol Classification System (Process Plant Engineers, Sheffield, UK) at 16 000 r min^−1^ to concentrate the cristobalite, which is found in greater abundance in the finer component (Horwell, Sparks *et al.*, 2003 ▶).

Crystalline silica was further concentrated from the respirable ash sample MRA5/6/99 by the Talvitie method (Talvitie, 1951 ▶), in which all minerals other than crystalline silica are dissolved by boiling the sample in phosphoric acid at 293 K for 8 min, and residues removed by washing with fluoroboric and hydrochloric acids. XRD revealed that the concentrated crystalline silica fraction was composed of 97 wt% cristobalite and 3 wt% quartz, although a trace of plagioclase feldspar (the dominant mineral phase in the bulk sample) remained, with perhaps also a trace of tridymite (Horwell, Sparks *et al.*, 2003 ▶). The residual plagioclase is likely to be due to the intimate association between cristobalite and other phases in individual ash particles (Horwell *et al.*, 2012 ▶).

### 2.3. Characterization of cristobalite and phase transitions   

Cristobalite in all five samples was identified using X-ray diffraction with a position-sensitive detector (XRD-PSD) at the Natural History Museum, London. This instrument consists of a Nonius X-ray diffraction system with an INEL curved 120° 2θ PSD with the beam and sample in static reflection geometry. We use the cristobalite peak of highest intensity (near 22° 2θ) for identification in our unconcentrated volcanic ash samples because lower-intensity cristobalite peaks are often masked by the presence of other minerals: plagioclase, in particular. XRD patterns were compared with the PDF-2 database cards from the ICDD (International Centre for Diffraction Data, http://www.icdd.com) for α- and β-cristobalite for identification purposes.

**Figure 1:**
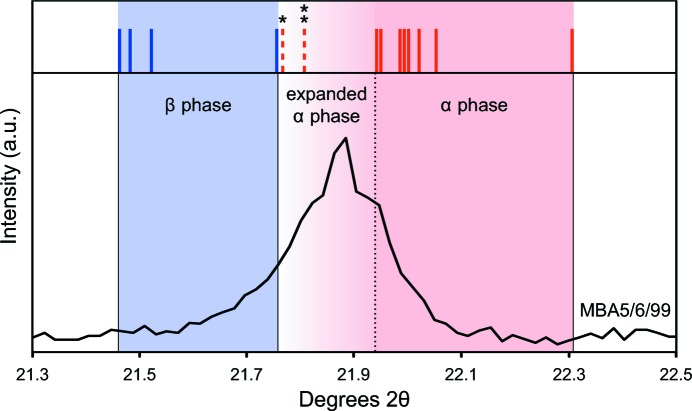
Range of (101)_α_ (red) and (111)_β_ (blue) cristobalite peak positions defined by ICDD library patterns. Vertical lines correspond to the peak positions of the 14 ICDD patterns under consideration. Dashed lines indicate patterns *01-082-0512 (structure with distorted ions) and **01-076-0941 (503 K). The diffraction profile of sample MBA5/6/99 is shown to demonstrate where volcanic cristobalite typically sits within these ranges.

To date, the ICDD database contains 14 theoretical and empirical powder diffraction patterns for cristobalite with sufficient information for comparison. These library patterns were overlaid using *WinX^POW^* (STOE, Darmstadt, Germany) in order to construct a database range for the 101 α- and 111 β-cristobalite peaks (see Fig. 1 ▶). The ICDD PDF-2 cards used for ambient α-cristobalite were 00-039-1425, 01-071-0785, 01-075-0923, 01-076-0935, 01-077-1316, 01-082-1232, 01-089-3434 and 01-089-3606. Two further PDF-2 cards for α-cristobalite were included: 01-082-0512, which is a crystal structure calculation for α-cristobalite with distorted ions, and 01-076-0941, which is a pattern collected at 503 K. The PDF-2 cards used for β-cristobalite were 01-085-0621, 01-089-3435, 01-089-3607 and 01-076-0934.

The α–β cristobalite phase transition in samples MBA5/6/99, MRA5/6/99 and Hemenway was investigated by XRD using a GeniX Cu high-flux X-ray system equipped with a Xenocs FOX two-dimensional 10_30P mirror to generate high-brightness Cu *K*α radiation (Natural History Museum, London). Samples were heated *in situ*, using an Anton Paar heating stage (HTK-10) with a platinum strip, from room temperature to 573 K with a stepwise heating profile at a rate of 10 K min^−1^. The high-brightness X-ray source allowed rapid data collection of isothermal diffraction patterns, each of only 1 min duration, at 2 K intervals. Thus, a sequential 0–120° 2θ snap-shot visualization of the changes in diffraction peak positions throughout the heating cycle was recorded by the PSD. The point at which the (101)_α_ peak began decreasing in intensity marked the onset temperature of the α–β transition, and the point at which the (101)_α_ peak became indistinguishable from background defined the end temperature. In order to monitor the effect of prolonged heating on the α–β transition and (101)_α_ *d*-spacing shift, additional heating experiments were performed, where samples were held at various mid-transition temperatures.

The position of the (101)_α_ cristobalite peak is affected by the overlap of a minor plagioclase peak, which appears as a shoulder on the cristobalite peak at higher 2θ angle; however, the plagioclase peak does not overlap with the (111)_β_ cristobalite peak. Thermal expansion of plagioclase with compositions typical of Soufriere Hills (An_>50_; Couch *et al.*, 2003 ▶) is relatively limited over the temperature variation in the present study, and most of the volume expansion is accommodated for anisotropically in the direction of the (100) plane normal (Tribaudino *et al.*, 2010 ▶); therefore, the plagioclase peak near 22.0° 2θ was considered to be unaffected by heating to 573 K. Consequently, it could be isolated from patterns collected at temperatures above the α–β transition and subtracted from the lower-temperature patterns, allowing the (101)_α_ cristobalite and plagioclase peaks to be deconvolved. Samples were left undisturbed during the entire heating sequence so that patterns for different temperatures could be directly compared. Since the plagioclase peak position remained fixed through consecutive runs, this is a good indication that no change in sample-stage height/sample surface occurred during the thermal heating experiments; therefore, no internal 2θ calibration standard was required and accurate peak positions were achieved using an external Si standard alone. Peak positions for the primary (101)_α_ and (111)_β_ cristobalite reflections are discussed according to the 2θ position at half-width half-maximum.

Differential scanning calorimetry (DSC) was used to provide a complementary determination of the α–β transition temperature for sample MBA5/6/99 observed by XRD. These results provide a transition-temperature range unobstructed by the plagioclase overlap as well as associated transition enthalpies for volcanic cristobalite. Samples were heated from 423 to 573 K (encompassing the entirety of the α–β transition range) on a PerkinElmer Pyris 1 differential scanning calorimeter in the Department of Chemistry, Durham University. The thermal stability of samples was assessed by thermogravimetric analysis (TGA) on a PerkinElmer Pyris 1 thermogravimetric analyser in the Department of Chemistry, Durham University.

### 2.4. Rapid quenching of volcanic β-cristobalite   

Samples of volcanic ash were heated through the cristobalite transition and cooled at different rates to investigate whether rapid cooling could preserve β-cristobalite. MBA5/6/99 was pressed into 10 mm pellets at 9.8 MPa, heated from ambient temperature to 573 K at 5 K min^−1^, and held for 1 h before being either rapidly quenched in liquid nitrogen or left to cool at room temperature. Pellets were gently ground and run immediately by XRD on a Bruker AXS D8 ADVANCE with DAVINCI design in 2θ reflection mode using Cu radiation and an Ni filter (Department of Chemistry, Durham University). Data were interrogated using the *DIFFRAC.EVA* software (Bruker AXS Inc., Madison, WA, USA). A control pellet was pressed (but not heated) and analysed by XRD under the same conditions.

### 2.5. Imaging and elemental composition of volcanic cristobalite   

Cross sections through cristobalite particles (MBA5/6/99) were prepared for analysis by mounting a sub-sample in resin (Araldite resin AY103 and hardener HY956, 5:1 ratio). The resin block was coated with 20 nm of carbon. Imaging and elemental analysis of cristobalite crystals were undertaken in a Hitachi SU-70 field emission gun scanning electron microscope equipped with an Oxford Instruments INCAx-act (LN_2_-free analytical silicon drift detector) energy-dispersive X-ray spectroscopy (EDS) system at the G. J. Russell Microscopy Facility, Durham University. Imaging was used to confirm the presence of the characteristic ‘fish-scale’ cracking in cristobalite and EDS was used to qualitatively confirm the presence of cation substitutions observed by Horwell *et al.* (2012 ▶).

## 3. Results   

### 3.1. Identification of volcanic cristobalite using library patterns   

A comprehensive comparison of ICDD library patterns for cristobalite revealed two distinct ranges for the α and β forms (Fig. 1 ▶). The primary (101)_α_ peak at ambient conditions is between *d*_101_ = 3.982 and 4.047 Å (21.9 to 22.3° 2θ). When considering the non-ambient (503 K) and distorted α-cristobalite patterns, the (101)_α_ peak range extends to *d*_101_ = 4.081 Å (21.8° 2θ). The primary (111)_β_ peak is between *d*_111_ = 4.082 and 4.137 Å (21.5 to 21.8° 2θ).

The *d* spacings observed at ambient temperature in this study for volcanic cristobalite were 4.064 (MBA5/6/99) and 4.067 Å (MRA5/6/99), and 4.057 Å after the Talvitie treatment (Table 1 ▶), which fit within the range for α-cristobalite identified from the ICDD patterns but are larger than the range compiled for α-cristobalite under ambient conditions. No evidence of β-cristobalite was observed in samples of volcanic ash at ambient temperature. The two synthetic cristobalite standards also fit within the range for α-cristobalite identified from the ICDD patterns: *d*_101_ = 4.055 Å (Hemen­way) and *d*_101_ = 4.038 Å (DKSmith).

### 3.2. Characterization of the displacive transition   

**Figure 2:**
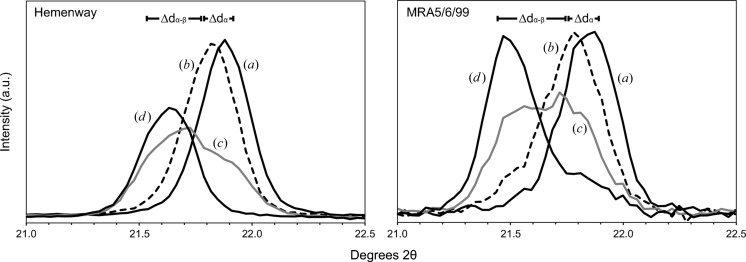
XRD patterns of the Hemenway cristobalite standard (left) and ash sample MRA5/6/99 (right) at four distinct temperatures: (*a*) room temperature as α-cristobalite; (*b*) prior to the α–β transition following thermal expansion (Δ*d*_α_); (*c*) midway through the transition; and (*d*) following the transition to β-cristobalite. Patterns were collected for intervals of 1 min per given temperature and are displayed between 21.0 and 22.5° 2θ to enlarge the primary (101)_α_ and (111)_β_ peak region.

Superimposed thermal XRD patterns at temperatures below, across and above the α–β transition from continuous runs for both volcanic cristobalite and the Hemenway synthetic sample show a clear change in the position of the primary cristobalite peak with increasing temperature, marking the α–β transition (Fig. 2 ▶). The transition occurs over a temperature range of around 40 K, taking place between 443 and 483 K in sample MBA5/6/99, 448 and 493 K in sample MRA5/6/99, and 513 and 553 K in the Hemenway sample. DSC data show a consistent, but slightly broader, temperature range of 448–508 K for the transition in volcanic sample MBA5/6/99.

Thermal XRD data show that the cristobalite transition comprises a gradual migration of the (101)_α_ peak position (Δ*d*_α_) towards lower 2θ angles, followed by a definitive change in *d* spacing at the α–β transition (Δ*d*_α–β_) (Fig. 2 ▶). For volcanic cristobalite, Δ*d*_α–β_ ranges from 0.046 to 0.051 Å. This change in primary peak position follows a Δ*d*_α_ of 0.08° 2θ (0.014 Å) from room temperature to the onset of the transition. These phenomena were also observed in the Hemenway sample, but with a smaller Δ*d*_α–β_ of 0.035 Å following a larger migration of Δ*d*_α_ of 0.018 Å.

**Figure 3:**
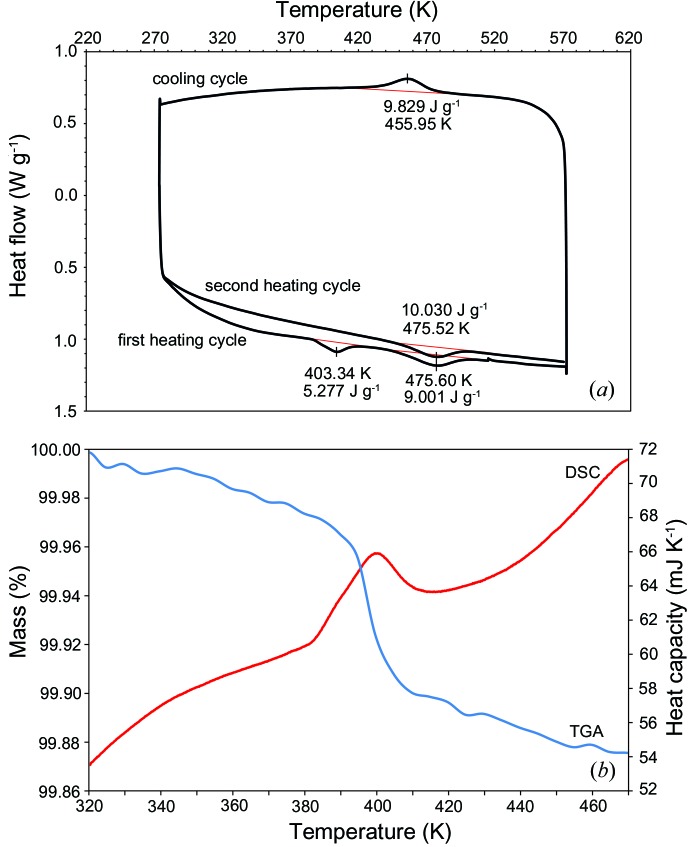
(*a*) The α–β transition of cristobalite detected by DSC (sample MBA5/6/99) with an initial heating cycle, a cooling cycle and a second heating cycle. The peaks at ∼475 K on heating and ∼456 K on cooling mark the phase transition. (*b*) Heat capacity of the anomalous peak centred on ∼403 K and corresponding mass loss measured by TGA. Scanning rate is 10 K min^−1^.

DSC data for MBA5/6/99 show two clear endothermic reactions during the first heating cycle, one at 403 K and another at 475 K (Fig. 3 ▶). Only one exothermic reaction took place on cooling, near 456 K, and only the higher-temperature transition recurred on reheating, indicating that the peak at 403 K is a nonreversible event (discussed in §[Sec sec4.5]4.5). As no other minerals identified in volcanic ash go through a transition in this temperature range, the mirrored reactions at 475 and 456 K are recording the transition from α- to β-cristobalite on heating and the reverse on cooling. The temperatures at which the phase transition occurs on heating and cooling do not correspond, however. The onset temperature of the transition during one cycle corresponds to the maximum transition temperature of the other cycle; *i.e.* the α to β transition on heating commences at the peak temperature of the cooling transition (449 K onset *versus* 456 K cooling cycle maximum), and *vice versa* for the β to α transition (475 K for both transition onset and heating cycle maximum) (Fig. 3 ▶). The transition shows an increased integrated heat flow for a consecutive structural rearrangement over two scans, from 9.001 to 10.030 J g^−1^ for MBA5/6/99 (Fig. 3 ▶). The susceptibility of cristobalite to thermal hysteresis explains the difference in the transition onset temperature upon heating and cooling.

The α–β phase transition proceeds as a function of temperature rather than time, as demonstrated when the transition was paused and the sample held isothermally mid-transition. An isothermal step maintained for 1 h upon heating through the transition range failed to progress the phase transition further; however, an additional increase in temperature following the isothermal step immediately resumed the inversion. When the temperature was held constant, the *d* spacing remained the same as that initially measured during the regular heating cycle. Additionally, the same proportion of α- and β-cristobalite was observed to exist at discrete temperatures in repeated experiments.

Cooling rate had no effect on the final crystal structure after first heating MBA5/6/99 to produce the β phase. No differences were observed in the cristobalite diffraction patterns for the non-heat-treated sample, the rapidly quenched sample and the sample slowly cooled to ambient conditions.

No change in mass was observed by TGA through the α–β transition range. However, a mass loss of ∼0.12 wt% was observed from 383 to 413 K (Fig. 3 ▶).

The peak positions for the Talvitie-treated separate differ from the corresponding non-treated sample MRA5/6/99 (*d*_α_ = 4.057 and 4.064 Å, respectively). This probably results from the boiling of the sample within the α–β transition temperature range in the Talvitie method. The sample was not subjected to thermal analysis since the treatment appears to have affected the sample’s thermochemistry, and further examination is, therefore, outside of the aims of this study. As a result, it is unknown whether intraparticle phases in volcanic ash identified by Horwell *et al.* (2012 ▶) impose a constraint on the cristobalite transition. However, data are included in Table 1 ▶ to show the effects of the concentration process on the crystal lattice.

### 3.3. Observation of cristobalite in ash particles   

**Figure 4:**
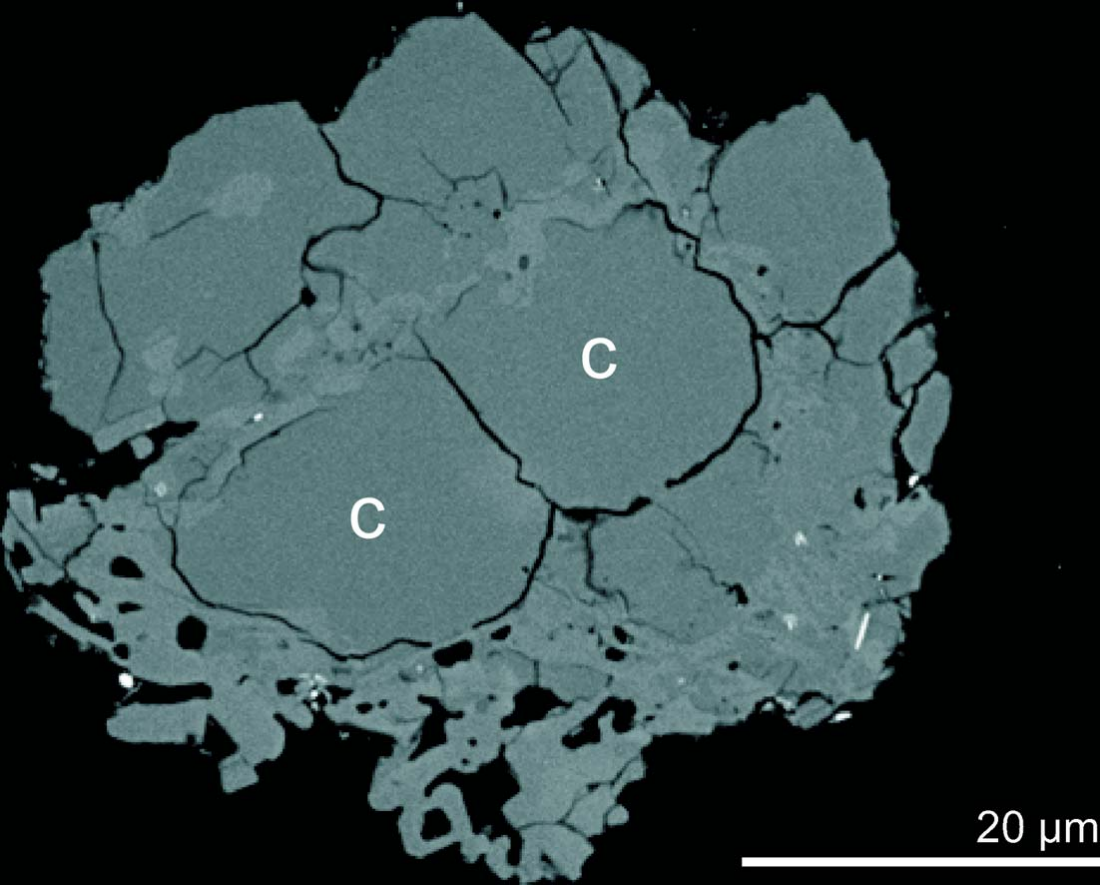
Backscattered electron image of a cristobalite-rich particle from ash sample MBA5/6/99 in polished section. Patches of cristobalite marked as ‘C’ are two ‘fish-scales’ characteristic of the ‘fish-scale’ cracking texture that occurs as cristobalite transforms from the β phase to the α phase on cooling. Lighter-grey patches are feldspar and glass. Image taken at 8.0 kV and working distance of 15.0 mm.

The distinctive ‘fish-scale’ texture was observed in cross sections through volcanic cristobalite (Fig. 4 ▶). Occasionally, individual crystals within ash grains did not display the ‘fish-scale’ texture, but these were always less than ∼8 µm in diameter and probably represent either a minimum crystal size for which the change in volume results in shattering or individual ‘fish-scales’ of cristobalite crystals that fragmented along the cracks during dome collapse. A critical grain size of >5 µm has been observed for which the thermally induced ‘fish-scale’ cracking can exist (Lee & Lee, 2000 ▶), which is appropriate for the present observations. Scanning electron microscopy (SEM)–EDS confirmed the presence of aluminium and sodium in cristobalite previously observed in sample MBA5/6/99 by Horwell, Sparks *et al.* (2003 ▶). The abundance of substitutions was not quantified in the present study, as previous analyses of cristobalite from Soufrière Hills have shown a range of 0.3–2.4 mol.% Al_2_O_3_ within distinct samples (Horwell *et al.*, 2012 ▶).

## 4. Discussion   

The presence of cristobalite with ‘fish-scale’ cracking in volcanic ash (Fig. 4 ▶) provides unequivocal evidence that it crystallized in the dome as β-cristobalite but subsequently transformed to the α form following eruption and cooling. Cristobalite with diagnostic cracking has also been observed in dome rock from Soufrière Hills (Horwell *et al.*, 2013 ▶; Plail *et al.*, 2014 ▶), Merapi (Damby *et al.*, 2013 ▶), Mount St Helens (Blundy & Cashman, 2001 ▶; Pallister *et al.*, 2008 ▶) and Santiaguito (Damby, 2012 ▶; Scott *et al.*, 2012 ▶), and sectioned ash from Sakurajima (Hillman *et al.*, 2012 ▶). In the following discussion we present evidence from experiments by thermal XRD (Fig. 2 ▶) and DSC (Fig. 3 ▶) on volcanic ash from Soufrière Hills to explain the dynamics of the total (excepting nanofibres, see §[Sec sec4.6]4.6) transition of β- to α-cristobalite during cooling.

### 4.1. Structure of volcanic cristobalite   

The interplanar spacings calculated by XRD for volcanic α-cristobalite (*d*_101_ = 4.064–4.067 Å) (Table 1 ▶) are larger than for the Hemenway sample (*d*_101_ = 4.055 Å), for the DKSmith sample (*d*_101_ = 4.038 Å), the range defined by ICDD patterns for α-cristobalite at ambient conditions and reported values for synthetic α-cristobalite with few impurity ions (*e.g.* *d*_101_ = 4.04 Å; Chao & Lu, 2002*a* ▶); they trend towards the (101)_α_ peak positions of the non-ambient and distorted ICDD α-cristobalite patterns. The larger *d* spacing observed in natural α-cristobalite probably results from the structural substitutions observed by Horwell *et al.* (2012 ▶). However, at elevated temperatures, the interplanar spacings of volcanic β-cristobalite (*d*_111_ = 4.097–4.124 Å) correspond well to those in the Hemenway sample at elevated temperature (*d*_111_ = 4.108 Å), as well as literature values for synthetic β-cristobalite (*d*_111_ = 4.11 Å) (Butler & Dyson, 1997 ▶) and the presently defined ICDD β-cristobalite range.

The peak widths for volcanic cristobalite are wider than those for the two synthetic cristobalite standards used, indicating a larger degree of disorder in the volcanic crystals and variable short-range interplanar spacings. A homogenous distribution of substituted cations would be expected to shift the 2θ diffraction peak profile whilst maintaining half-peak width to height ratios; however, Damby (2012 ▶) reports a heterogeneous distribution of substituted ions throughout individual cristobalite crystals. Such chemical variation will result in non-uniform *d* spacings and lead to a broader XRD peak profile, as is observed, and supports the conclusion of structurally incorporated cations in volcanic cristobalite. Poorly ordered cristobalite results in an XRD pattern that has a higher background and decreased sharpness and intensity of reflections compared to a well ordered sample (Alcalá *et al.*, 1996 ▶); therefore, we suggest that structural disorder needs to be carefully considered when quantifying mineral phases by peak height [*e.g.* the IAS method of Le Blond *et al.* (2009 ▶)].

Caution should also be taken when determining the presence of α *versus* β polymorphs in volcanic ash on the basis of primary peak matching with the ICDD database. Such matching could potentially prove to be ambiguous (Fig. 1 ▶), which we attribute to three factors: (1) the increased lattice spacing of (Al,Na)-bearing cristobalite in volcanic ash samples, which broadens and shifts the (101)_α_ diffraction peak towards the position where the (111)_β_ peak occurs; (2) the ubiquitous presence in cristobalite-bearing volcanic ash of an overlapping plagioclase peak near 22° 2θ, which masks the true position of the cristobalite peak; and (3) the range in the ICDD peak positions reported from different laboratories. These factors can lead to the primary cristobalite peak in volcanic ash often appearing closer to certain library patterns for β-cristobalite rather than α-cristobalite. Additionally, it is possible that user-based differences in sample preparation, instrument deployment and instrument calibration methods can result in different peak positions that are of variable accuracy.

Notwithstanding these issues, the ranges for α and β forms defined by the ICDD library patterns may help with identification by eliminating the need to deal with the aforementioned considerations. For example, in the study by Keith & Muffler (1978 ▶), which identified both α- and β-cristobalite in tuff deposits, the (101)_α_ peak was quite sharp and ranged from 21.8 to 22.0° 2θ_Cu_ (*d*_101_ = 4.07–4.04 Å), whereas the (111)_β_ peak was usually rounded and ranged from 21.6 to 21.8° 2θ_Cu_ (*d*_111_ = 4.11–4.07 Å). These ranges are in good agreement with the present study.

### 4.2. Characterization of the α–β cristobalite transition   

The cristobalite inversion involves a transition enthalpy, which is recorded by the endo- and exothermic reactions observed by DSC (Fig. 3 ▶). No other major phase in these samples identified by XRD undergoes a first-order transition between 443 and 513 K, leaving cristobalite as the only discrete phase inversion in this range. The diffraction intensity and lattice-spacing data also confirm a structural inversion of cristobalite in Soufrière Hills volcanic ash (Δ*d*_α–β_ = 0.046–0.051 Å). The transition-temperature ranges observed by DSC (448–508 K) and XRD (443–483 K) are in close agreement and probably differ because of the different instrument heating/temperature measurement techniques and the manner in which transition temperatures were determined from the raw data.

Comparing XRD patterns from prior to and after a heating and cooling cycle demonstrates that there is no change in cristobalite peak intensity or in the proportions of other phases present. Therefore, from the characteristics of the (101)_α_ peak, volcanic cristobalite reverts back to the original disordered isomorph upon cooling. This reversion of disordered α-cristobalite to the same defect structure following a transition was also observed by Butler & Dyson (1997 ▶) and probably results from the inability of foreign ions and vacancies that stabilize the disordered form to be substantially varied on the timescale of the experiments. Further, repeat analyses on sub-samples show that the same proportions of α- and β-cristobalite coexist at a specific temperature. Therefore, the increase in the integrated heat flow from subsequent runs of the same sample (Fig. 3 ▶) does not necessarily imply an increase in cristobalite abundance or inherent structural order, but instead is likely to result from the irreversible nature of the transition hysteresis for cristobalite, where cycling through the α–β transition is accompanied by an increase in entropy (Richet *et al.*, 1982 ▶).

The mixed-phase nature of volcanic ash makes it difficult to relate the heat of transition for the α–β inversion to that observed for pure-phase synthetic samples. However, in scaling up the enthalpy associated with the transition in sample MBA5/6/99, which has ∼11 wt% cristobalite on the basis of an XRD quantification by Horwell *et al.* (2014 ▶), the heat flow for a ‘pure-phase’ sample of volcanic cristobalite would be approximately 5400 J mol^−1^. This result is in poor agreement with values from previous studies on synthetic samples, of 2320 and 1300 J mol^−1^ (Thompson & Wennemer, 1979 ▶; Mosesman & Pitzer, 1941 ▶), especially since less energy is required to transform a disordered cristobalite sample (with a larger unit cell) compared with a better ordered sample (Butler & Dyson, 1997 ▶). We note, however, that the quantification by Horwell *et al.* (2014 ▶) has an uncertainty of ±3 wt%, which is problematic given that a difference of 1 wt%, from 11 to 10 wt%, would result in ∼500 J mol^−1^ difference in heat flow. This effect scales with the proportion of cristobalite and, therefore, the error in calculated heat flow will increase with increasing cristobalite abundance. Further, as a mixed-phase dust, sub-samples of volcanic ash are expected to contain variable quantities of mineral phases; therefore, it is not possible to confirm the abundance of cristobalite in the sub-sample used for the DSC measurements. As such, more accurate quantification of cristobalite in heterogeneous dusts would be required to constrain the effects of structural defects on the transition energy.

### 4.3. Controls on the α–β transition temperature   

The α–β transition temperature for Soufrière Hills ash occurs over a broader temperature range than is generally reported for synthetic cristobalite samples: ∼40 K for ash *versus* ∼20–40 K for synthetic samples (Leadbetter & Wright, 1976 ▶; Mosesman & Pitzer, 1941 ▶; Sosman, 1965 ▶). We propose that the breadth of the transition reflects heterogeneity amongst cristobalite crystal grains, resulting from the presence of substituted ions and associated structural defects. Cristobalite crystallization can commence within hours or days of a magma package entering the dome environment (Williamson *et al.*, 2010 ▶; Horwell *et al.*, 2014 ▶), and crystals will be subjected to variable temperatures, pressures and interaction with hydrothermal fluids throughout their growth and residence. As a result of this dynamic thermochemical environment, volcanic cristobalite probably undergoes multiple phases of disordered growth and ion substitution. As discussed in §[Sec sec1.2]1.2, these factors can increase the transition breadth compared with a homogenous cristobalite crystal population (Thompson & Wennemer, 1979 ▶; Stevens *et al.*, 1997 ▶).

The transition temperature for the cristobalite in our ash samples (443–483 K) falls at the low end of the range reported for synthetic cristobalite samples (§[Sec sec1.2]1.2) and is much lower than that in our Hemenway sample (513–553 K). We find that volcanic cristobalite exhibits a lower degree of thermal expansion (Δ*d*_α_) than synthetic cristobalite, but expansion for both samples begins at similar temperatures: ∼403 K for volcanic cristobalite and 393 K for the Hemenway sample. Consequently, we hypothesize that the lower α–β inversion temperature observed in volcanic cristobalite may result from a smaller Δ*d*_α_ associated with thermal expansion prior to the transition to β-cristobalite. This phenomenon would be facilitated by substituted and interstitial cations in volcanic cristobalite. O atoms in tetrahedra surrounding an Al^3+^ substitution will be under-bonded and any such O atoms also adjacent to an interstitial Na^+^ (satisfying local charge balance) are likely to occupy a slightly larger thermal ellipsoid and be dynamically (or statically) displaced from their preferred tetrahedral geometry within such a defect region. If the thermal energy input was transferred with higher probability to the O atoms local to impurity (Al^3+^, Na^+^) defect regions relative to other O atoms in the framework, then this may result in an increased thermal ellipsoid vibration for the O atoms incorporated at defects. In such a situation, the absorption of thermal energy primarily by increased oxygen vibrations in defect regions together with a more limited overall lattice expansion may be a sufficient response to cope with an incremental increase in thermal energy. Conversely, the lattice of α-cristobalite with few substitutions/defects may behave more uniformly and expand evenly to a greater overall degree in response to applied heat. In this way, local expansion and oxygen displacement in defect regions may trigger the α–β transition by lowering the activation energy.

Cristobalite in the ash sample is likely to have formed through both vapour-phase mineralization and groundmass devitrification (Baxter *et al.*, 1999 ▶; Horwell *et al.*, 2012 ▶); a potential for these two mechanisms to produce crystals with different Al abundances has been reported (Damby, 2012 ▶). The above discussion would therefore suggest a potential difference in the α–β transition temperature for cristobalite of vapour-phase and devitrification origin. However, we cannot test this hypothesis here because it was not possible to obtain separate samples for vapour-phase and devitrification cristobalite.

In synthetic cristobalite samples, lower transition temperatures are associated with lower crystallization temperatures (§[Sec sec1.2]1.2). The volcanic dome environment is typically below ∼1073 K (Barclay *et al.*, 1998 ▶; Murphy *et al.*, 2000 ▶), which is more than 300 K cooler than the minimum crystallization temperatures used to synthesize cristobalite; for example, a minimum temperature of 1373 K was used in experiments by Alcalá *et al.* (1996 ▶) and Stevens *et al.* (1997 ▶). By assuming a crystallization temperature of less than 1273 K in a volcanic setting, and extrapolating the observed effects in synthetic samples annealed between 1273 and 1673 K to cooler temperatures, we hypothesize that the lower inversion temperature seen for volcanic cristobalite may partially reflect the crystallization temperature in the dome.

### 4.4. Coexistence of α and β phases   

It is apparent from the continuous DSC endo- and exotherms (Fig. 3 ▶) and the gradual emergence of the (111)_β_ peak and disappearance of the (101)_α_ peak during thermal XRD (Fig. 2 ▶) that α and β forms coexist during the phase transition. This observation agrees with studies on synthetic cristobalite (*e.g.* Leadbetter & Wright, 1976 ▶; Swainson *et al.*, 2003 ▶; Schmahl *et al.*, 1992 ▶; Spearing *et al.*, 1992 ▶), whereby a smooth change in the intensity of these modes is observed as the proportion of cristobalite existing as either α or β varies with temperature (§[Sec sec1.2]1.2). The coexistence interval is larger in volcanic samples than has been observed for synthetic samples; two potentially complementary factors could contribute to the observed coexistence interval (§[Sec sec1.2]1.2): cation substitution and crystallite size.

The range of cation substitutions observed in volcanic cristobalite crystals, from 0.30 to 2.38 mol.% Al_2_O_3_ (Horwell *et al.*, 2012 ▶), may support the existence of different transition temperatures amongst grains, as the amount of β-cristobalite retained metastably in experimental studies is known to depend on the level of additives (Chao & Lu, 2002*b* ▶). Domains with a higher degree of disorder/abundance of additive ions are likely to transform at lower temperatures than purer regions. Since the temperature and duration of heating in these experiments are insufficient to allow significant diffusion of additives, it might be expected that the same transformation behaviour, in terms of coexisting α/β proportions and the ranges of transformation temperature observed on cooling and re-heating, will be replicated exactly if the impurity regions are those where the transformation (*via* oxygen displacement) is initiated.

The broad temperature range of the α–β transition could also result from polydisperse crystal sizes in our samples, where individual crystallites transform at different temperatures (§[Sec sec1.2]1.2). A heterogeneous cristobalite grain size distribution has been quantitatively observed for volcanic dome samples, where larger crystals can form in domes that have longer residence times (Damby, 2012 ▶). A grain size control may be manifest in the small difference between XRD and DSC transition ranges as only the XRD samples were ground in a mortar and pestle prior to analysis.

### 4.5. Anomalous DSC peak   

The endothermic reaction in the initial DSC heating cycle at 403 K is a nonreversible event as no corresponding event took place upon cooling (Fig. 3 ▶). Most likely, this event is a dehydration reaction, resulting either from surface hydration and open porosity or from the crystallization of an amorphous/hydrous phase such as opal-C or opal-CT. These two phenomena are indistinguishable in the current data as powdered opal requires minimal or no diffusion of ‘free’ water to the surface and, therefore, water is lost at a temperature below that of a corresponding lump sample (Smallwood *et al.* 2008 ▶). Opal-C can be indistinguishable from cristobalite by XRD since the characteristic 101 peak around 21° 2θ (4.00–4.10 Å) of these materials coincides (*e.g.* Onal *et al.*, 2007 ▶), although opal-C usually produces a broad diffraction peak compared with cristobalite. Considering a Δ*H* of vaporization at 403 K of 39.1 kJ mol^−1^, this corresponds to 0.24 wt% of water in the sample, which is larger than the 0.12 wt% observed by TGA. This theoretical mass loss could correspond to as much as 5 wt% opal-C in the sample. The difference between the theoretical water content and experimental TGA data could be explained by a variation in mineral abundance in sub-samples from a single sample: we frequently observe variability of a few weight per cent for other phases in volcanic ash. This amount of opal-C (or opal-CT) would be near background level in the thermal XRD patterns as 60 s isothermal data collection snapshots resulted in peaks with intensities of only ∼1000 counts.

Alternatively, the discontinuity observed during the first heating cycle may reflect a tridymite inversion, if this phase is present at low abundance (not detectable by XRD) in the cristobalite. λ transitions in tridymite are observed near 288 K in synthetic tridymite–cristobalite mixed phases (Thompson & Wennemer, 1979 ▶; Stevens *et al.*, 1997 ▶). However, as all tridymite phase transitions are reversible (Graetsch & Flörke, 1991 ▶), a discontinuous inversion from tridymite to cristobalite would be required as no corresponding structural rearrangements are seen on further heating and cooling cycles. The change in latent heat observed in the first and second heating cycles (9.001 J g^−1^ for the first heating cycle *versus* 10.03 J g^−1^ for the second) could be interpreted as evidence of the transformation of tridymite to cristobalite, with incorporation of its associated latent heat into the subsequent cristobalite inversion.

### 4.6. Preservation of β-cristobalite and implications for exposure   

From textural studies (Horwell *et al.*, 2013 ▶) and the heating experiments contained herein, cristobalite crystallizes and persists in the volcanic dome in the β form when above the α–β transition temperature. However, during cooling, the charge-coupled substitutions in volcanic cristobalite are insufficient to suppress the transition to α-cristobalite as is possible with chemically stabilized cristobalite in the Na_2_O–Al_2_O_3_–SiO_2_ system (*e.g.* Chao & Lu, 2002*b* ▶; Perrotta *et al.*, 1989 ▶). As discussed in §[Sec sec1.2]1.2, a maximum of 2.4 mol.% Al_2_O_3_ was observed in volcanic cristobalite, whereas stabilization of β-cristobalite in synthetic samples required a minimum of 6.29 mol.% Al_2_O_3_. Furthermore, the observed crystals are much too large for nanoscale effects (§[Sec sec1.2]1.2) to suppress the β to α transition for the bulk of cristobalite in volcanic ash. However, we propose that the preservation of β-cristobalite nanofibres observed by transmission electron microscopy in ash from the Chaitén volcano, Chile (Reich *et al.*, 2009 ▶), is an effect of grain size, since the diameter of the fibres (∼20–50 nm) is below the length scale (∼50 nm) at which nanoscale phase-transition effects have been observed (McKnight *et al.*, 2008 ▶).

These observations, combined with our failure to preserve β-cristobalite in the ash through rapid quenching experiments, imply that cristobalite in volcanic ash is overwhelmingly Al-bearing α-cristobalite following an eruption; hence, this is the phase of relevance to human exposure. From the present data, we conclude that α-cristobalite standards are appropriate to assess the toxicity of volcanic ash from a polymorphic perspective, although perhaps not with regards to structure (or composition, as shown by Horwell *et al.*, 2012 ▶). However, if a small proportion of nearly amorphous hydrous silica is responsible for the anomalous DSC endotherm, this may have ramifications for toxicity as the bioreactivity will differ between cristobalite and other arrangements of SiO_2_ (*e.g.* Pavan *et al.*, 2013 ▶).

## 5. Conclusion   

This is the first structural and thermodynamic investigation of volcanic cristobalite and exploits the α–β phase transition to determine its characteristics. Volcanic cristobalite commonly exhibits substitutions, mainly Al^3+^ and Na^+^ for Si^4+^ (Horwell *et al.*, 2012 ▶), which serve to increase the size of the unit cell and to lower and broaden the α–β transition temperature relative to more pure SiO_2_ samples. Both thermal XRD and DSC demonstrate that α-cristobalite exists in volcanic ash, indicating that these substitutions are insufficient to stabilize β-cristobalite at ambient temperature, as has been achieved with higher levels of Al and Na co-substitutions in chemically stabilized synthetic cristobalite. However, the observed structural alteration can lead to confusion when indexing against ICDD library patterns owing to a shift in the primary (101)_α_ peak to lower angles. The data presented here may help to clear up complexities shown by inverted samples and the broad range in the ICDD database; to facilitate this, we have defined distinct *d*-spacing ranges for α- (21.8–22.5° 2θ) and β-cristobalite (21.5–21.7° 2θ) that could be used in the future for identification.

Upon heating, volcanic cristobalite exhibits a smaller degree of thermal expansion (Δ*d*_α_) of the (101)_α_ planar spacing prior to the α–β transition compared with synthetic samples. Since the onset of this thermal expansion occurs at similar temperatures for both volcanic and synthetic cristobalite, we suggest that the smaller Δ*d*_α_ in volcanic cristobalite may be a direct result of the degree of substituted and interstitial ions, which also effectively cause the α–β transition to occur at lower temperatures. Owing to peak overlaps in mixed-phase volcanic ash, three-dimensional expansion could not be considered. No differences in β-cristobalite cell dimensions were observed compared with standard cristobalite samples or accepted values from studies of synthetic cristobalite.

Since crystalline silica from different environments has been shown to be a variable respiratory hazard in industrial settings, constraining the volcanic cristobalite hazard is imperative for hazard response during an eruptive episode. The verification of α-cristobalite in volcanic ash means that toxicity studies which compare volcanic cristobalite with α-cristobalite standards are valid. However, natural cristobalite will always show some cation substitutions, and the hazard posed by exposure to respirable volcanic cristobalite should be considered accordingly.

## Figures and Tables

**Table 1:** Summary of *d* spacings and transition parameters for samples investigated Lattice data are in ångström and temperatures are in kelvin. *d*(101)_α_ and *d*(111)_β_ are the *d* spacings for primary α- and β-cristobalite peaks, respectively. Δ*d*_α_ is the thermal expansion of *d*(101)_α_ for the α phase prior to the onset of the α–β transition. Δ*d*_α–β_ is the change in the *d* spacing during the transition following thermal expansion (Δ*d*_α_). *T*_tr_ is the transition temperature range for the α–β inversion detected by XRD and DSC.

Sample	Type	*d*(101)_α_ 298 K	*d*(111)_β_ 573 K	Δ*d*_α_	Δ*d*_α–β_	*T*_tr_ (XRD)	*T*_tr_ (DSC)
MBA5/6/99	Ash	4.064	4.124	0.014	0.046	443–483	448–508
MRA5/6/99	Ash	4.067	4.133	0.015	0.051	448–493	–
Hemenway	Standard	4.055	4.108	0.018	0.035	513–553	–
DKSmith	Standard	4.038	–	–	–	–	–
Talvitie	Isolate	4.057	–	–	–	–	–

## References

[bb1] Alcalá, M. D., Real, C. & Criado, J. M. (1996). *J. Am. Ceram. Soc.* **76**, 1681–1684.

[bb2] Barclay, J., Rutherford, M. J., Carroll, M. R., Murphy, M. D., Devine, J. D., Gardner, J. & Sparks, R. S. J. (1998). *Geophys. Res. Lett.* **25**, 3437–3440.

[bb3] Baxter, P. J., Bonadonna, C., Dupree, R., Hards, V. L., Kohn, S. C., Murphy, M. D., Nichols, A., Nicholson, R. A., Norton, G., Searl, A., Sparks, R. S. J. & Vickers, B. P. (1999). *Science*, **283**, 1142–1145. 10.1126/science.283.5405.1142 10024235

[bb4] Beckett, W. (2000). *New Engl. J. Med.* **342**, 406–413. 10.1056/NEJM200002103420607 10666432

[bb200] Blundy, J. & Cashman, K. (2001). *Contrib. Miner. Petrol.* **140**, 631–650.

[bb5] Brodie, K. H. & Rutter, E. H. (2000). *Geophys. Res. Lett.* **27**, 3089–3092.

[bb6] Butler, M. A. & Dyson, D. J. (1997). *J. Appl. Cryst.* **30**, 467–475.

[bb7] Carpenter, M. A., Salje, E. K. H. & Graeme-Barber, A. (1998). *Eur. J. Mineral.* **10**, 621–691.

[bb8] Chao, C.-H. & Lu, H.-Y. (2002*a*). *Mater. Sci. Eng. A*, **328**, 267–276.

[bb9] Chao, C.-H. & Lu, H.-Y. (2002*b*). *Metall. Mater. Trans.* **33**, 2703–2711.

[bb10] Clouter, A., Brown, D., Höhr, D., Borm, P. & Donaldson, K. (2001). *Toxicol. Sci.* **63**, 90–98. 10.1093/toxsci/63.1.90 11509748

[bb11] Couch, S., Hartford, C. L., Sparks, R. S. J. & Carrol, M. R. (2003). *J. Petrol.* **44**, 1455–1475.

[bb12] Cullen, R. T., Jones, A. D., Miller, B. G., Donaldson, K., Davis, J. M. G., Wilson, M. & Tran, C. L. (2002). Report TM/02/01. Institute of Occupational Medicine, Edinburgh, UK.

[bb13] Damby, D. E. (2012). Durham University e-thesis 1–258, Durham, UK.

[bb14] Damby, D. E., Horwell, C. J., Baxter, P. J., Delmelle, P., Donaldson, K., Dunster, C., Fubini, B., Murphy, F., Nattrass, C., Sweeney, S., Tetley, T. D. & Tomatis, M. (2013). *J. Volcanol. Geotherm. Res.* **261**, 376–387.

[bb15] Donaldson, K. & Borm, P. J. A. (1998). *Ann. Occup. Hyg.* **42**, 287–294. 10.1016/s0003-4878(98)00044-1 9729916

[bb16] Duffin, R., Gilmour, P. S., Schins, R. P. F., Clouter, A., Guy, K., Brown, D. M., MacNee, W., Borm, P. J., Donaldson, K. & Stone, V. (2001). *Toxicol. Appl. Pharmacol.* **176**, 10–17. 10.1006/taap.2001.9268 11578144

[bb17] Fenoglio, I., Fubini, B., Tiozzo, R. & Di Renzo, F. (2000). *Inhalation Toxicol.* **12**, 81–89. 10.1080/08958378.2000.11463233 26368603

[bb18] Forbes, L., Jarvis, D., Potts, J. & Baxter, P. (2003). *Occup. Environ. Med.* **60**, 207–211. 10.1136/oem.60.3.207 PMC1740482 12598669

[bb19] Frondel, C. (1962). *The System of Mineralogy: Silica Minerals*, 7th ed. New York: Wiley.

[bb20] Fubini, B., Bolis, V., Cavenago, A. & Volante, M. (1995). *Scand. J. Work Environ. Health*, **21**, 9–14. 8929680

[bb21] Graetsch, H. & Flörke, O. W. (1991). *Z. Kristallogr.* **195**, 31–48.

[bb22] Guthrie, G. D. Jr (1997). *Environ. Health Perspect.* **105**, 1003–1011. 10.1289/ehp.97105s51003 PMC1470178 9400692

[bb23] Hillman, S. E., Horwell, C. J., Densmore, A. L., Damby, D. E., Fubini, B., Ishimine, Y. & Tomatis, M. (2012). *Bull. Volcanol.* **74**, 913–930.

[bb24] Horwell, C. J. & Baxter, P. J. (2006). *Bull. Volcanol.* **69**, 1–24.

[bb25] Horwell, C. J., Fenoglio, I., Ragnarsdottir, K. V., Sparks, R. S. J. & Fubini, B. (2003). *Environ. Res.* **93**, 202–215. 10.1016/s0013-9351(03)00044-6 12963405

[bb26] Horwell, C. J., Hillman, S. E., Cole, P. D., Loughlin, S. C., Llewellin, E. W., Damby, D. E. & Christopher, T. (2014). *Controls on Variations in Cristobalite Abundance in Ash Generated by the Soufrière Hills Volcano, Montserrat in the Period 1997–2010*, Geological Society Memoir No. 39, pp. 399–406. Geological Society of London.

[bb27] Horwell, C. J., Sparks, R. S. J., Brewer, T. S., Llewellin, E. W. & Williamson, B. J. (2003). *Bull. Volcanol.* **65**, 346–362.

[bb28] Horwell, C. J., Williamson, B. J., Donaldson, K., Le Blond, J. S., Damby, D. E. & Bowen, L. (2012). *Part. Fibre Toxicol.* **9**, 44. 10.1186/1743-8977-9-44 PMC3574026 23164071

[bb29] Horwell, C. J., Williamson, B. J., Llewellin, E. W., Damby, D. E. & Le Blond, J. S. (2013). *Bull. Volcanol.* **75**, 1–19.

[bb30] IARC (1997). *Silica, Some Silicates, Coal Dust and Para-aramid Fibrils*, IARC Monographs on the Evaluation of Carcinogenic Risks to Humans, Vol. 68, pp. 1–475. International Agency for Research on Cancer. PMC5366849 9303953

[bb31] Jones, T. & BéruBé, K. (2011). *J. Hazad. Mater.* **194**, 128–134. 10.1016/j.jhazmat.2011.07.092 21872393

[bb32] Keith, T. E. C. & Muffler, L. J. P. (1978). *J. Volcanol. Geotherm. Res.* **3**, 373–402.

[bb33] Keith, T. E. C., White, D. E. & Beeson, M. H. (1978). *Hydrothermal Alteration and Self-Sealing in Y-7 and Y-8 Drill Holes in Northern Part of Upper Geyser Basin, Yellowstone National Park, Wyoming*, US Geological Survey Professional Paper 1054-A, A1–A26. Washington, DC: US Government Printing Office.

[bb35] Leadbetter, A. J. & Wright, A. F. (1976). *Philos. Mag.* **33**, 105–112.

[bb34] Le Blond, J. S., Cressey, G., Horwell, C. J. & Williamson, B. J. (2009). *Powder Diffraction*, **24**, 17–23.

[bb36] Lee, S. J. & Lee, C. H. (2000). *Mater. Lett.* **45**, 175–179.

[bb38] McKnight, R. E. A., Moxon, T., Buckley, A., Taylor, P. A., Darling, T. W. & Carpenter, M. A. (2008). *J. Phys. Condens. Matter*, **20**, 075229. 10.1088/0953-8984/22/3/035406 21386289

[bb39] Mosesman, M. A. & Pitzer, K. S. (1941). *J. Am. Chem. Soc.* **63**, 2348–2356.

[bb40] Mossman, B. T. & Glenn, R. E. (2013). *Crit. Rev. Toxicol.* pp. 1–29. 10.3109/10408444.2013.818617 23863112

[bb41] Murphy, M. D., Sparks, R. S. J., Barclay, J., Carroll, M. R. & Brewer, T. S. (2000). *J. Petrol.* **41**, 21–42.

[bb42] Napierska, D., Thomassen, L. C. J., Lison, D., Martens, J. A. & Hoet, P. H. (2010). *Part. Fibre Toxicol.* **7**, 39. 10.1186/1743-8977-7-39 PMC3014868 21126379

[bb43] Onal, M., Kahraman, S. & Sarikaya, Y. (2007). *Appl. Clay Sci.* **35**, 25–30.

[bb201] Pallister, J. S., Thornber, C. R., Cashman, K. V., Clynne, M. A., Lowers, H. A., Mandeville, C. W., Brownfield, I. K. & Meeker, G. P. (2008). *A Volcano Rekindled; the Renewed Eruption of Mount St Helens, 2004–2006*, US Geological Survey Professional Paper 1750, pp. 647–702.

[bb44] Pavan, C., Tomatis, M., Ghiazza, M., Rabolli, V., Bolis, V., Lison, D. & Fubini, B. (2013). *Chem. Res. Toxicol.* **26**, 1188–1198. 10.1021/tx400105f 23819533

[bb45] Perrotta, A. J., Grubbs, D. K., Martin, E. S., Dando, N. R., McKinstry, H. A. & Huarg, C.-Y. (1989). *J. Am. Ceram. Soc.* **72**, 441–447.

[bb46] Pertsev, N. A. & Salje, E. K. H. (2000). *Phys. Rev. B*, **61**, 902–908.

[bb47] Plail, M., Edmonds, M., Humphreys, M. C. S., Barclay, J. & Herd, R. A. (2014). *Earth Planet. Sci. Lett.* **386**, 21–33.

[bb48] Reich, M., Zúñiga, A., Amigo, Á., Vargas, G., Morata, D., Palacios, C., Parada, M. Á. & Garreaud, R. D. (2009). *Geology*, **37**, 435–438.

[bb49] Richet, P., Bottinga, Y., Denielou, L., Petitet, J. P. & Tequi, C. (1982). *Geochim. Cosmochim. Acta*, **46**, 2639–2658.

[bb50] Rios, S., Salje, E. K. H. & Redfern, S. A. T. (2001). *Eur. Phys. J. B*, **20**, 75–83.

[bb51] Schmahl, W. W. (1993). *Eur. J. Mineral.* **5**, 377–380.

[bb52] Schmahl, W. W., Swainson, I. P., Dove, M. T. & Graeme-Barber, A. (1992). *Z. Kristallogr.* **201**, 125–145.

[bb53] Scott, J. A. J., Mather, T. A., Pyle, D. M., Rose, W. I. & Chigna, G. (2012). *J. Volcanol. Geotherm. Res.* **237–238**, 54–68.

[bb54] Smallwood, G., Thomas, P. S. & Ray, A. S. (2008). *J. Thermal Anal. Calorim.* **92**, 91–95.

[bb55] Sosman, R. B. (1965). *The Phases of Silica.* New Brunswick: Rutgers University Press.

[bb56] Spearing, D. R., Farnan, I. & Stebbins, J. F. (1992). *Phys. Chem. Miner.* **19**, 307–321.

[bb57] Stevens, S. J., Hand, R. J. & Sharp, J. H. (1997). *J. Therm. Anal.* **49**, 1409–1415.

[bb58] Stone, V., Jones, R., Rollo, K., Duffin, R., Donaldson, K. & Brown, D. M. (2004). *Toxicol. Lett.* **149**, 255–259. 10.1016/j.toxlet.2003.12.036 15093271

[bb59] Swainson, I. P., Dove, M. T. & Palmer, D. C. (2003). *Phys. Chem. Miner.* **30**, 353–365.

[bb60] Talvitie, N. A. (1951). *Anal. Chem.* **23**, 623–631.

[bb61] Thomas, E. S., Thompson, J. G., Withers, R. L., Stern, M., Xiao, Y. & Kirkpatrick, R. J. (1994). *J. Am. Ceram. Soc.* **77**, 49–56.

[bb62] Thompson, A. B. & Wennemer, M. (1979). *Am. Mineral.* **64**, 1018–1026.

[bb63] Tribaudino, M., Angel, R. J., Ca’mara, F., Nestola, F., Pasqual, D. & Margiolaki, I. (2010). *Contrib. Mineral. Petrol.* **160**, 899–908.

[bb64] Wahl, F. M., Grim, R. E. & Graf, R. B. (1961). *Am. Mineral.* **46**, 1064–1076.

[bb65] Williamson, B. J., Di Muro, A., Horwell, C. J., Spieler, O. & Llewellin, E. W. (2010). *Earth Planet. Sci. Lett.* **295**, 83–90.

[bb66] Wilson, M. R., Stone, V., Cullen, R. T., Searl, A., Maynard, R. L. & Donaldson, K. (2000). *Occup. Environ. Med.* **57**, 727–733. 10.1136/oem.57.11.727 PMC1739881 11024195

[bb67] Xu, H., Heaney, P. J. & Navrotsky, A. (2001). *Phys. Chem. Miner.* **28**, 302–312.

